# Corona Discharge and Field Electron Emission in Ambient Air Using a Sharp Metal Needle: Formation and Reactivity of CO_3_^−•^ and O_2_^−•^

**DOI:** 10.5702/massspectrometry.A0100

**Published:** 2021-12-25

**Authors:** Kenzo Hiraoka, Stephanie Rankin-Turner, Satoshi Ninomiya, Haruo Shimada, Kazumasa Kinoshita, Shinichi Yamabe

**Affiliations:** 1 Clean Energy Research Center, University of Yamanashi, 4–3–11 Takeda, Kofu, Yamanashi 400–8511, Japan; 2 W. Harry Feinstone Department of Molecular Microbiology & Immunology, Johns Hopkins Bloomberg School of Public Health, Johns Hopkins University, Baltimore, MD, 21205, USA; 3 Graduate Faculty of Interdisciplinary Research, University of Yamanashi, 4–3–11 Takeda, Kofu, Yamanashi 400–8511, Japan; 4 Bio Chromato, Inc., 1–12–19 Honcho, Fujisawa, Kanagawa 251–0053, Japan; 5 Department of Chemistry, Nara University of Education, Takabatake-cho, Nara 630–8528, Japan

**Keywords:** CO_3_^−•^, H^•^ abstraction, HCO_3_^−^, O_2_^−•^, tunneling electron emission

## Abstract

CO_3_^−•^ and O_2_^−•^ are known to be strong oxidizing reagents in biological systems. CO_3_^−•^ in particular can cause serious damage to DNA and proteins by H^•^ abstraction reactions. However, H^•^ abstraction of CO_3_^−•^ in the gas phase has not yet been reported. In this work we report on gas-phase ion/molecule reactions of CO_3_^−•^ and O_2_^−•^ with various molecules. CO_3_^−•^ was generated by the corona discharge of an O_2_ reagent gas using a cylindrical tube ion source. O_2_^−•^ was generated by the application of a 15 kHz high frequency voltage to a sharp needle in ambient air at the threshold voltage for the appearance of an ion signal. In the reactions of CO_3_^−•^, a decrease in signal intensities of CO_3_^−•^ accompanied by the simultaneous increase of that of HCO_3_^−^ was observed when organic compounds with H–C bond energies lower than ∼100 kcal mol^−1^ such as *n*-hexane, cyclohexane, methanol, ethanol, 1-propanol, 2-propanol, and toluene were introduced into the ion source. This clearly indicates the occurrence of H^•^ abstraction. O_2_^−•^ abstracts H^+^ from acid molecules such as formic, acetic, trifluoroacetic, nitric and amino acids. Gas-phase CO_3_^−•^ may play a role as a strong oxidizing reagent as it does in the condensed phase. The major discharge product CO_3_^−•^ in addition to O_2_^−•^, O_3_, and NO*_x_^•^* that are formed in ambient air may cause damage to biological systems.

## INTRODUCTION

Basic data on gas-phase and condensed-phase reactions of CO_3_^−•^ and O_2_^−•^ are of importance in aeronomy, environmental chemistry, biology, and medicine. In Scheme 1, the mechanisms for the formation of CO_3_^−•^, HCO_3_^−^, and NO_3_^−^ *via* consecutive gas-phase ion/molecule reactions originating from O_2_, H_2_O, and N_2_ are summarized. In gas phase reactions of CO_3_^−•^, O^−•^ transfer reactions with the formation of CO_2_ were found to be the major reaction channels for NO, NO_2_, SO_2_,^1)^ N_2_O_5_,^2)^ and 2,4,6-trinitrotoluene.^3)^ Fehsenfeld *et al.*^4)^ and van der Linde *et al.*^5)^ reported that CO_3_^−•^ reacted with HNO_3_ *via* a proton transfer reaction to form NO_3_^−^ and HCO_3_^•^. In addition, van der Linde *et al.*^6)^ studied gas-phase reactions of CO_3_^−•^ with formic acid (HCOOH) to form [HCOO^−^····OH^•^] using FT-ICR mass spectrometry. Ninomiya *et al.*^3)^ predicted that CO_3_^−•^ reacts with H_2_O_2_ to form the cluster ion, O_2_^−•^····H_2_CO_3_. As of this writing, however, no H^•^ abstraction by CO_3_^−•^ in the gas phase has been reported, even though H^•^ abstraction reactions are a major concern due to its potential for causing damage in biological systems.^7)^ Thus, it would be of interest to examine the issue of whether CO_3_^−•^ also abstracts H^•^ from organic compounds in the gas-phase. Kawashima *et al.* measured product ions formed from collisionally excited cluster ions of [CO_3_^−•^+M] in the gas phase with M being 16 amino acids and organic acids.^8)^ They detected HCO_3_^−^ as the major product ion formed from amino acids. This indicates the occurrence of H^•^ abstraction by CO_3_^−•^ in collisionally excited cluster ions of [CO_3_^−•^+M]*. Their results may give some insight into the subject of H^•^ abstraction reactions of CO_3_^−•^ in the gas phase.

**Figure:**
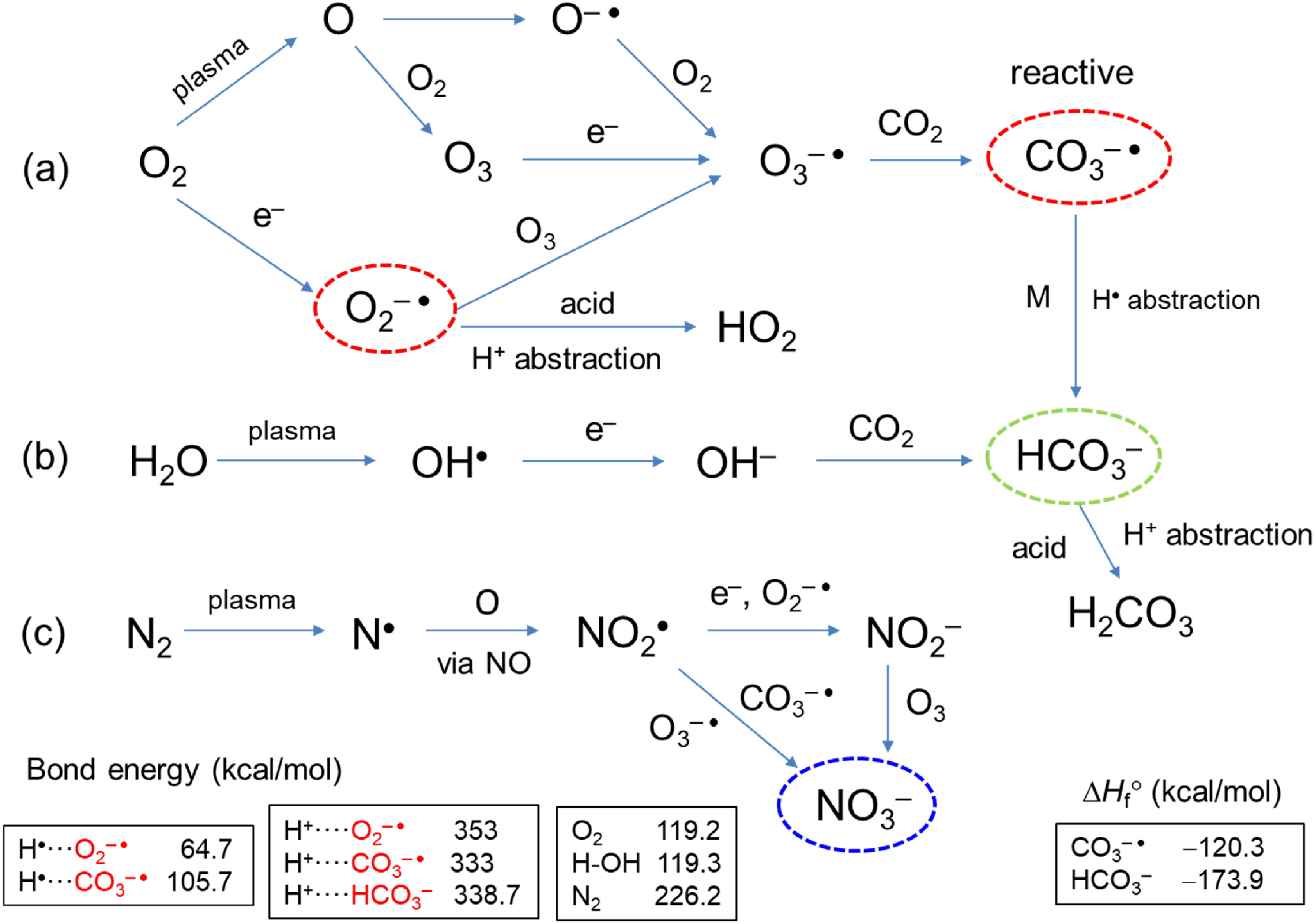
Scheme 1. Consecutive reactions of plasma-excited air starting from O_2_ (a), H_2_O (b), and N_2_ (c).

In reactions of CO_3_^−•^ in aqueous solutions, Elango *et al.*^9)^ reported that CO_3_^−•^ reacted with aliphatic amines by (i) a H^•^ abstraction to form HCO_3_^−^ and (ii) an electron transfer to form CO_3_^2−^ (one electron oxidation). The former is more probable in cases of primary amines, while tertiary amines reacted *via* electron transfer. Clifton and Huie^10)^ measured rate constants in aqueous solutions for H^•^ abstraction reactions of CO_3_^−•^ with several saturated alcohols and cyclic ethers. The Arrhenius pre-exponential factors ranged from 2×10^8^ to 1×10^9^ M^−1^s^−1^ and the activation energies ranged from 3.8 to 6.9 kcal mol^−1^ (1 cal=4.18 J). Crean *et al.*^11)^ investigated the oxidation of single-stranded oligonucleotides by CO_3_^−•^, leading to the generation of intrastranded cross-links between guanine and thymine bases that were separated by cytosines. Roginskaya *et al.*^12)^ studied the efficacy and site specificity of H^•^ abstraction from DNA 2-deoxyribose by CO_3_^−•^ and also evaluated the selectivity of damage in double-stranded DNA. Karmakar and Datta^13)^ reported on the reactivity of CO_3_^−•^ for six amino acid chains (Cys, Met, Phe, Tyr, His and Trp) using state-of-the-art density functional theory. They reported that CO_3_^−•^ causes oxidative damage to amino acid residues predominantly *via* H^•^ abstraction with moderate to high rate constants.

Various types of gas-phase ion/molecule reactions of O_2_^−•^ have also been investigated, including [1] S_N_2 (nucleophilic second-order substitution) reactions for CH_3_Br,^14)^ CH_3_Cl,^14)^ CF_3_CO_2_CH_3_,^14)^ and CH_3_CO_2_CH_3_,^14)^ [2] charge (electron) transfer reactions for CCl_2_F_2_,^15)^ CCl_3_F,^15)^ SF_6_,^16–18)^ 2,4,6-trinitrotoluene,^19)^ and O_3_,^20)^ [3] H^+^ abstraction reactions for CF_3_SO_3_H,^21)^ HCl,^21,22)^ FSO_3_H,^23)^ and HNO_3_,^4)^ and [4] clustering reactions for CH_3_CN,^23)^ (CF_3_)_2_CO,^14)^ H_2_C=CHCN,^14)^ (CH_3_)_2_CO,^14)^ and higher hydrocarbons.^24)^

In a physiological environment, the superoxide anion O_2_^−•^ can function as an oxidant or a reductant, and the dismutation reaction, 2O_2_^−•^+2H^+^ → H_2_O_2_+O_2_, is an example of this.^7)^ The reaction (1) of O_2_^−•^ with NO^•^ has received special attention due to the fact that peroxynitrite, ONOO^−^, a strong biological oxidant, is generated. 
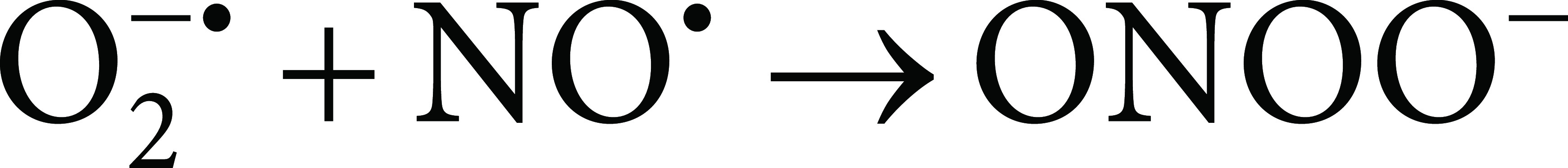
(1)

Peroxynitrite reacts with CO_2_ to produce CO_3_^−•^ *via* reactions (2) and (3). 
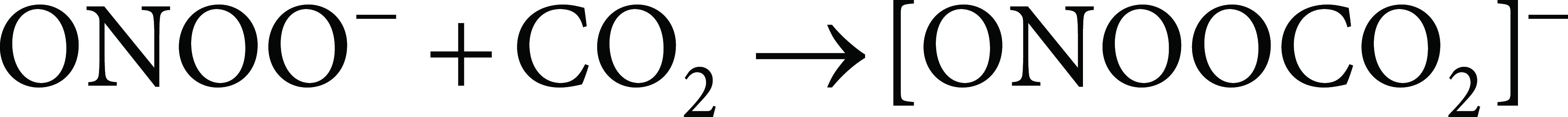
(2)
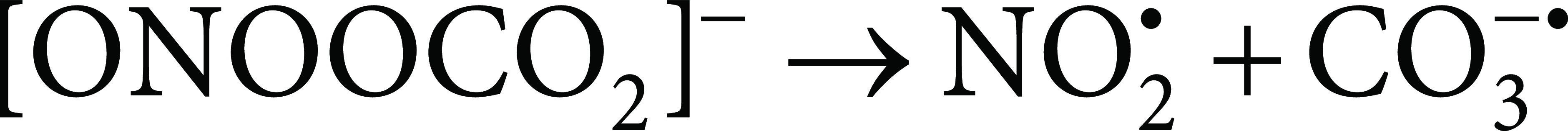
(3)

That is, O_2_^−•^ triggers the formation of CO_3_^−•^, which is a highly oxidative species in biological systems.

In the present study, the gas-phase reactions of CO_3_^−•^ and O_2_^−•^ with various organic molecules such as hydrocarbons, alcohols, and acids were investigated. It was observed that CO_3_^−•^ abstracts H^•^ from methanol, ethanol, 1-propanol, 2-propanol, *n*-hexane, cyclohexane, and toluene, to form HCO_3_^−^. In the reaction of CO_3_^−•^ with H_2_O_2_, O_2_^−•^····H_2_CO_3_ cluster ions were detected. In contrast, the only type of reaction for O_2_^−•^ observed in this experiment was H^+^ abstraction reactions from various acid molecules (*i.e.*, formic acid, acetic acid, nitric acid, trifluoroacetic acid, and amino acids).

## EXPERIMENTAL

### Materials

Reagent grade *n*-hexane, cyclohexane, benzene, toluene, methanol, ethanol, 1-propanol, 2-propanol, acetone, acetonitrile, formic acid, acetic acid, trifluoroacetic acid, nitric acid, and amino acids (leucine, isoleucine, alanine, and phenylalanine) were purchased from FUJIFILM Wako Pure Chemical (Osaka, Japan). CO_2_ gas (liquefied CO_2_, 99.9%, Nitto-Bussan Corp., Yamanashi, Japan) and O_2_ gas (ZERO-U, >99.999%, Sumitomo Seika Chemicals Corp., Tokyo, Japan) were used as reagent gases.

### Ion source assembly for the formation of CO_3_^−•^

**Figure 1:**
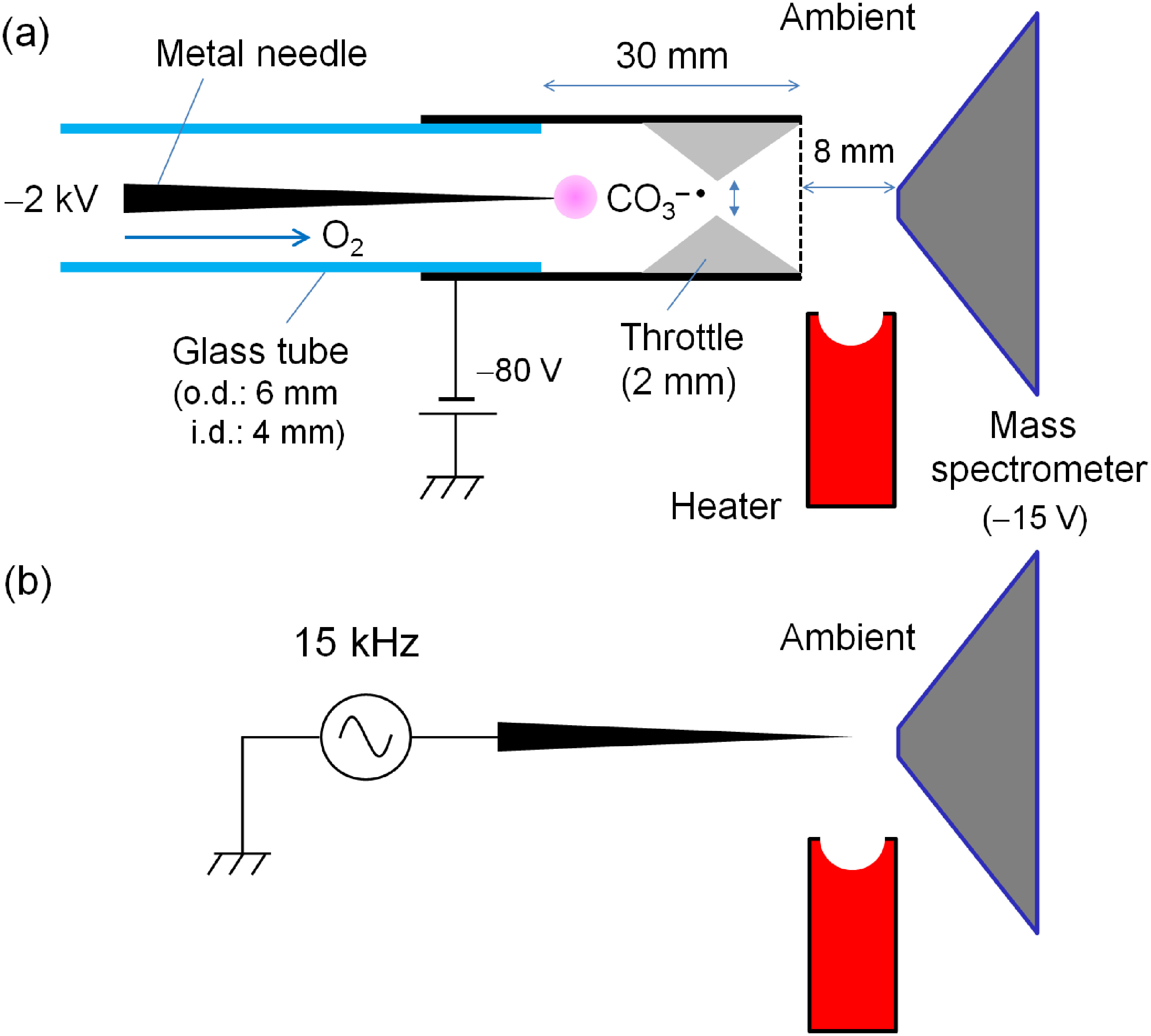
Fig. 1. (a) Direct current (DC) corona discharge ion source for the formation of CO_3_^−•^ using O_2_ as the reagent gas. (b) 15 kHz alternating current (AC) voltage applied to the needle electrode in ambient air.

**Figure 2:**
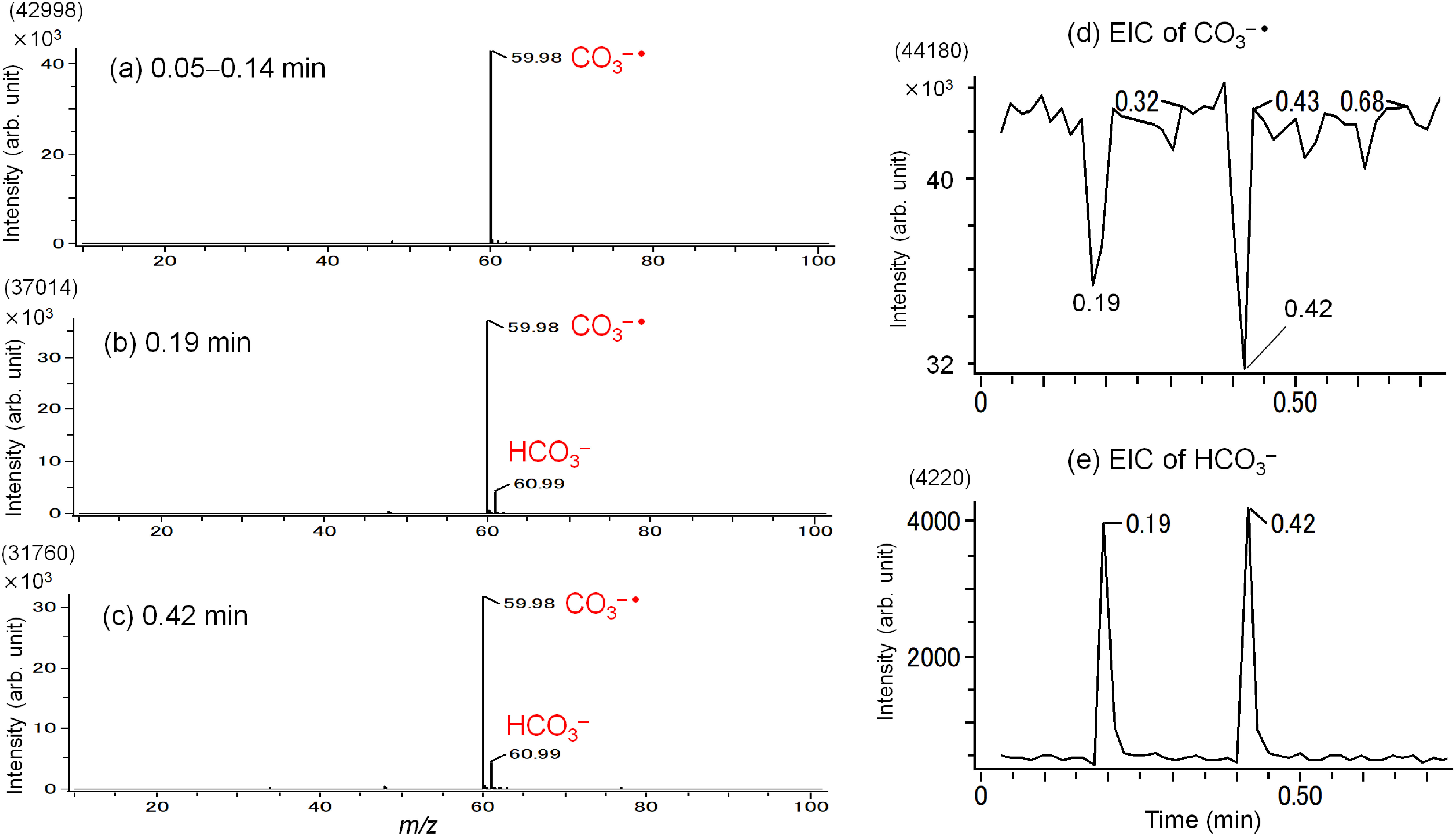
Fig. 2. (a) Mass spectrum before sample introduction. (b) Mass spectrum when *n*-hexane is introduced into the ion source at 0.19 min. (c) Mass spectrum when *n*-hexane is introduced into the ion source at 0.42 min. (d) EIC of CO_3_^−•^ (*m*/*z* 60). (e) EIC of HCO_3_^−^ (*m*/*z* 61).

The mass spectrometric measurements were performed with a time-of-flight mass spectrometer (AccuTOF, JEOL, Akishima, Tokyo, Japan). Figure 1(a) shows the assembly of the cylindrical ion source tube that was used for the formation of CO_3_^−•^. The ambient air open distance between the terminal end of the cylindrical tube and the ion sampling orifice of the mass spectrometer was 8 mm. When CO_2_ was used as the reagent gas for the formation of CO_3_^−•^, O_2_^−•^ with a relative intensity of about 20–30% compared to CO_3_^−•^ was unavoidable, as shown in Fig. S1. The formation of O_2_^−•^ may originate from O_2_ contamination in the CO_2_ reagent gas or O_2_ formed by the decomposition of the CO_2_ reagent gas in the corona discharge plasma. In contrast, when O_2_ was used instead of CO_2_ as the reagent gas, CO_3_^−•^ was generated as the major ion and the formation of O_2_^−•^ was negligible (see Fig. 2(a)). This indicates that O_3_^−•^ produced by the DC discharge of the reagent O_2_ gas was efficiently converted into CO_3_^−•^. The source of the carbon for the formation of CO_3_^−•^ may be the impurity of CO_2_ in the O_2_ reagent gas, adsorbed CO_2_ and/or CO on the wall of the gas pipe line.

The use of a stainless steel throttle shown in Fig. 1(a) is essential for the generation of CO_3_^−•^ as the major ion. When the throttle was not used, the back diffusion of air into the inside of the tube could not be avoided, even when the tip of the needle electrode was recessed a distance of 10 mm from the exit of the glass tube. The back diffusion of air was readily recognized by the detection of HCO_3_^−^ originating from the moisture in the air (see Scheme 1(b)). When the tip of the needle electrode was recessed a distance of 30 mm from the exit of the mesh-covered glass tube with the throttle inserted near the exit, the back diffusion of air was nearly completely avoided and CO_3_^−•^ ions were formed as the only major reactant ion flowing out of the glass tube. Thus, the back diffusion of reactant vapor introduced into the plasma region of the ion source appears to be negligible in the present experimental setup.

The O_2_ reagent gas with a flow rate of 3 L min^−1^ was ionized by a direct current (DC) corona discharge. The consecutive reactions (4)–(11) took place in ambient air (Scheme 1(a)). The major ion O_2_^−•^ that was generated was formed by an electron attachment reaction (4), then reacted with O_3_ (major neural product in corona discharge of O_2_ reagent gas) to form O_3_^−•^ by the charge transfer reaction (10). O_3_^−•^ further reacted with the CO_2_ impurity in the O_2_ carrier gas to form CO_3_^−•^ (reaction (11)). 
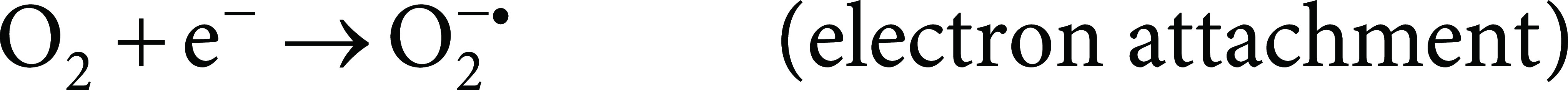
(4)
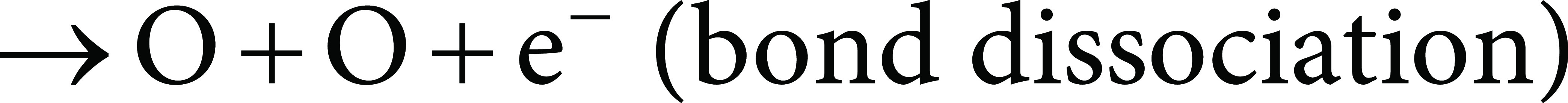
(5)
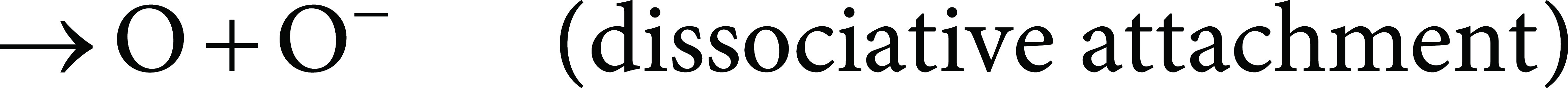
(6)
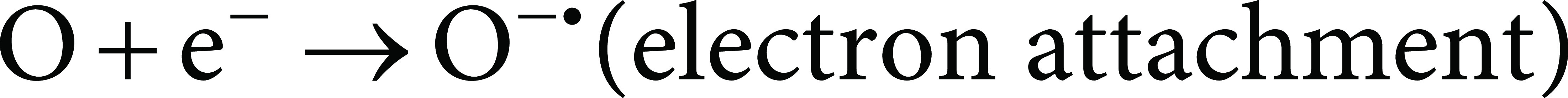
(7)
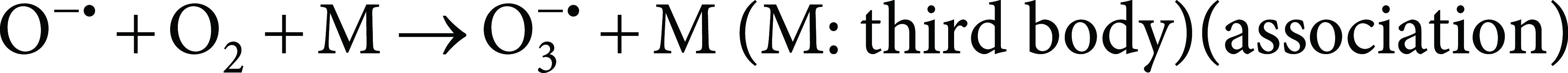
(8)
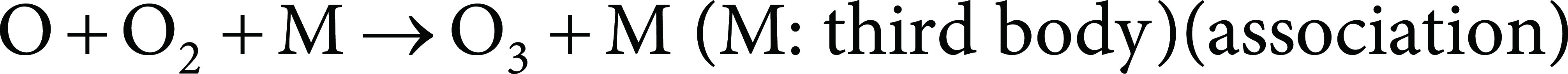
(9)
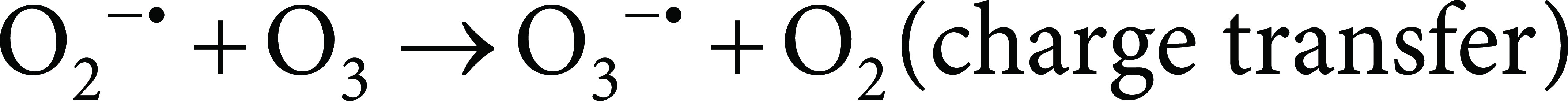
(10)
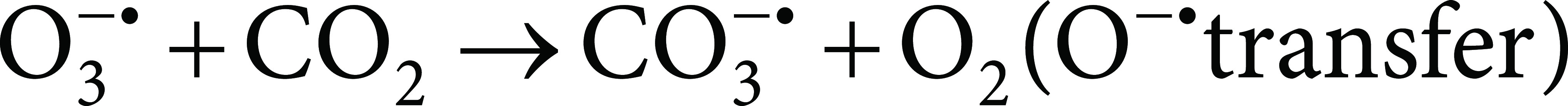
(11)

The changes in the enthalpy for reactions (10) and (11) were calculated to be −37.6^19)^ and −11.8^25)^ kcal mol^−1^, respectively, and reactions (10) and (11) proceed with nearly collision rates.^23)^

### O_2_^−•^ formation using a sharp metal needle in ambient air

The simple experimental setup for this process is shown in Fig. 1(b). The formation of O_2_^−•^ as the major ion was only observed when a high-frequency (15 kHz) threshold voltage for the appearance of the ion signal was applied to the needle in ambient air. This phenomenon is attributed to the field electron emission from the tip of the sharp needle (see the latter section).

## RESULTS AND DISCUSSION

### Reactions of CO_3_^−•^ with various molecules

By using the ion source shown in Fig. 1(a), reactions of CO_3_^−•^ with hydrocarbons (*n*-hexane, cyclohexane, benzene, and toluene), alcohols (methanol, ethanol, 1-propanol, and 2-propanol), acetonitrile, acetone, water, and H_2_O_2_ were examined. A 10 μL aliquot of a liquid sample was placed on the well of the heater shown in Fig. 1. The heater temperature was maintained at a temperature of about 30°C above the boiling point of the liquid.

As an example, experimental results obtained for *n*-hexane are shown in Fig. 2. Figure 2(a) shows the mass spectrum before sample introduction, in which the only detected ion was CO_3_^−•^. Figures 2(b) and 2(c) show the mass spectra that were obtained when 10 μL liquid hexane was placed on the heater twice at 0.19 min and at 0.42 min. On the introduction of the sample, HCO_3_^−^ (*m*/*z* 61) appeared at 0.19 min and at 0.42 min, respectively. Figures 2(d) and 2(e) show the extracted ion current (EIC) chronograms for CO_3_^−•^ and HCO_3_^−^, respectively. A sharp decrease in CO_3_^−•^ and an increase in HCO_3_^−^ were simultaneously observed when the sample was introduced into the ion source. These results clearly indicate that CO_3_^−•^ abstracts H^•^ from *n*-C_6_H_14_, with the formation of HCO_3_^−^ in the gas phase (reaction (12)). 
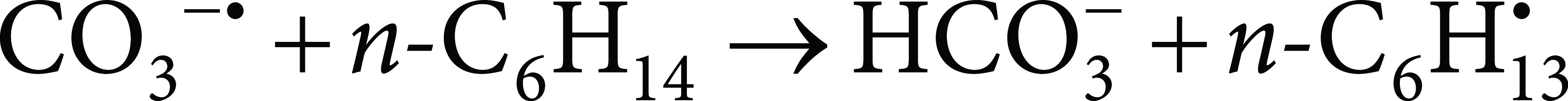
(12)

Gas-phase H^•^ abstraction reactions by CO_3_^−•^ have not been extensively explored, which may be due to the fact that reactant molecules are not detected as ions in H^•^ abstraction reactions. In this work, H^•^ abstraction reactions were also detected for cyclohexane, toluene, methanol, ethanol, 1-propanol, and 2-propanol (data not shown). However, H_2_O, benzene, acetone, and acetonitrile did not show any noticeable reactivity toward CO_3_^−•^ under the present experimental conditions.

From the value for the heat of formation of HCO_3_^−^ (−173.9 kcal mol^−1^),^26)^ the bond energy of H^•^····CO_3_^−^ was estimated to be 105.7 kcal mol^−1^. Table S1 summarizes the H–C bond energies of hydrocarbons and alcohols and that of H–O bond of H_2_O.^25,26)^ Roughly speaking, CO_3_^−•^ abstracts H^•^ from molecules that have H–C bond energies equal to or smaller than ∼100 kcal mol^−1^. In one exceptional case, the ion intensity of CO_3_^−•^ did not change and HCO_3_^−^ was not detected when acetonitrile (bond energy of H–C bond of methyl group: 93.2 kcal mol^−1^) was introduced into the ion source. At present, we have no explanation for why CO_3_^−•^ did not show any noticeable reactivity toward acetonitrile, but there may be a substantial entropy barrier for this reaction. In the present work, occurrence/non-occurrence experiments on the reactivity of CO_3_^−•^ in the gas phase were conducted for the first time, though no quantitative information such as rate constants was obtained.

In order to determine whether or not CO_3_^−•^ can abstract a hydrogen atom from organic molecules (Ms), DFT calculations were carried out for the reaction of CO_3_^−•^ with three representative molecules, namely, benzene, *n*-hexane and toluene. The computational method is M06-2X/6-311++G(2p,d), which gives very accurate thermochemical data and activation energies.^27)^ Using this approach, the geometries of the transition state (TS) were determined. Subsequently, the intrinsic reaction coordinate (IRC) was traced to obtain the geometries of weakly bound complexes, CO_3_^−•^····M and HCO_3_^−^+[M−H]^•^. Gaussian 16 was used for all calculations.^28)^ Fig. S2 exhibits the Braumann-type energy changes expressed by differences in electronic and zero-point vibrational energies. Fig. S3 shows TS geometries. In Fig. S2, the reaction for benzene has a large endoergicity, +14.60 kcal/mol, for the formation of HCO_3_^−^+[M−H]^•^ and can be ruled out since it is in agreement with the experimental results. In contrast, for *n*-hexane and toluene, H^•^ abstraction reactions are likely to occur. Spin densities were calculated for the “HCO_3_^−^····[M−H]^•^” complex, and it was confirmed that the reaction involves the abstraction of a hydrogen atom and does not produce HCO_3_^•^····[M−H]^−^ (*i.e.*, no proton transfer). The occurrence of H^•^ abstraction reactions may be due to the fact that the energy barrier of the transition state is of the same order as the bond energies of the ion–molecule complexes. In addition, the tunneling effect of H^•^ may contribute to these reactions, *i.e.*, the wave matter of H^•^ penetrates through the barrier without crossing the barrier.

**Figure 3:**
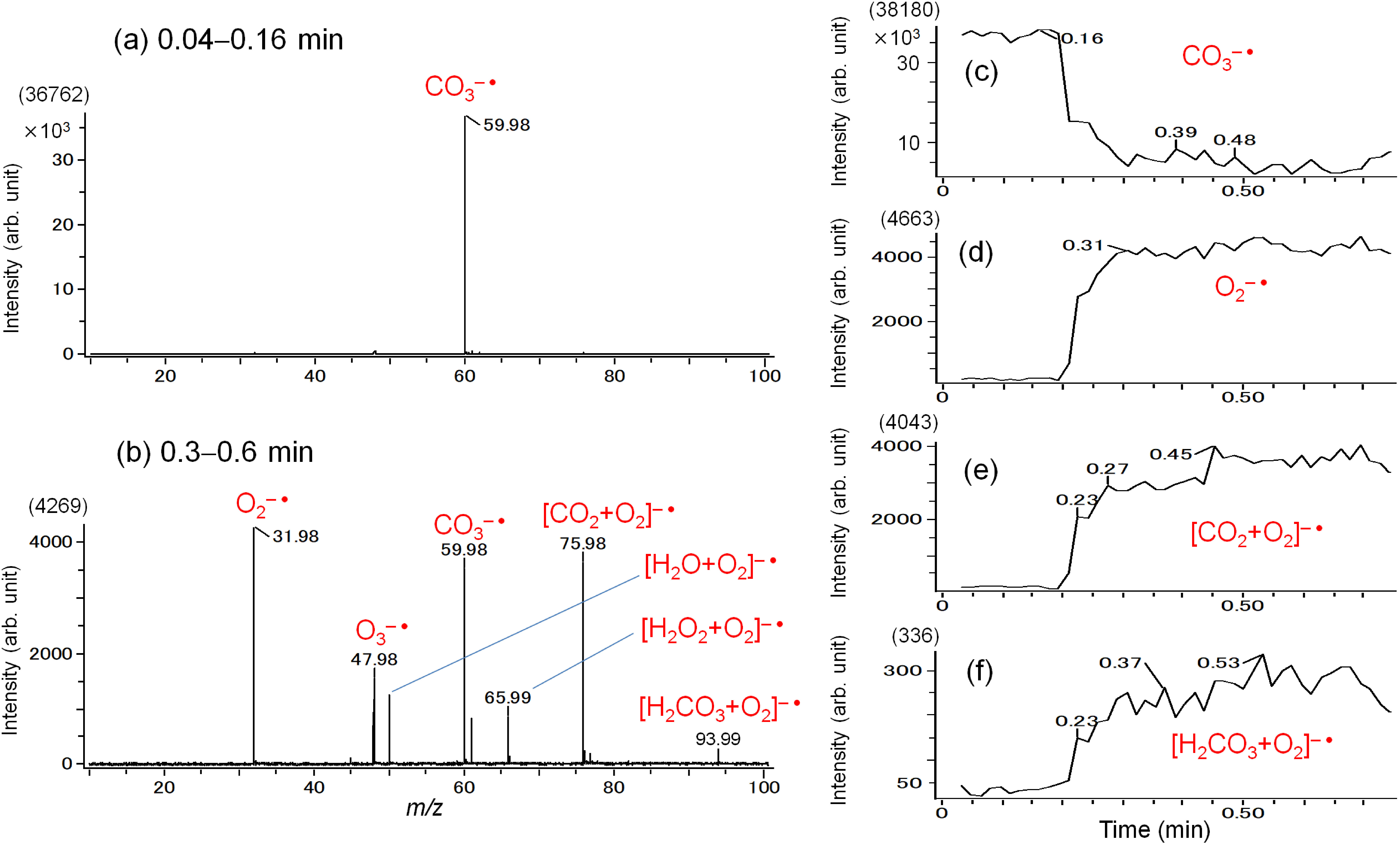
Fig. 3. (a) Mass spectrum before sample introduction. (b) Mass spectrum showing the introduction of H_2_O_2_ into the ion source. (c) EIC of CO_3_^−•^ (*m*/*z* 60). (d) EIC of O_2_^−•^ (*m*/*z* 32). (e) EIC of [CO_2_+O_2_]^−•^ (*m*/*z* 76). (f) EIC of [H_2_CO_3_+O_2_]^−•^ (*m*/*z* 94). 10 μL of 30% aqueous solution of H_2_O_2_ was placed in the well of the heater at 120°C.

In our previous work,^3)^ based on theory, we predicted that CO_3_^−•^ reacts with H_2_O_2_ to form O_2_^−•^····H_2_CO_3_ cluster ions. However, this cluster ion was not detected experimentally in our previous study.^3)^ This may be due to the relatively low abundance of CO_3_^−•^ generated by the ambient-air corona discharge. Figures 3(a) and 3(b) show the mass spectra before and after the introduction of H_2_O_2_ into the ion source shown in Fig. 1(a), respectively. In Fig. 3(b), the cluster ion O_2_^−•^····H_2_CO_3_ (*m*/*z* 94) in addition to the base peak for O_2_^−•^ was clearly detected. The cluster ion O_2_^−•^····H_2_CO_3_ may be formed by the collisional stabilization of the intermediate complex, [O_2_^−•^····H_2_CO_3_]*. 
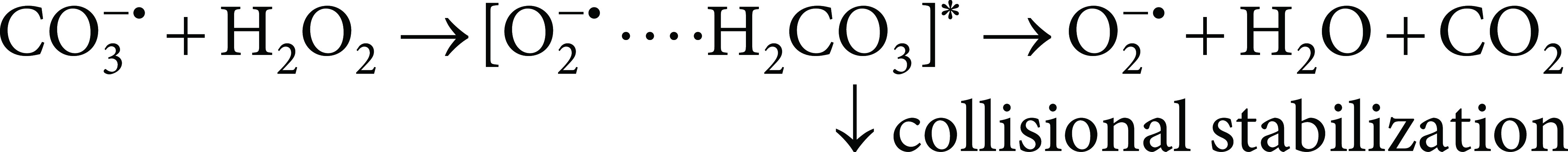
(13-1)
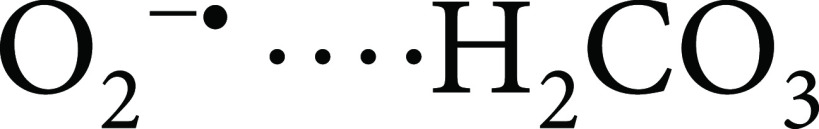
(13-2)

The cluster ion of O_2_^−•^····H_2_CO_3_ detected in this work suggests that the unimolecular decomposition of H_2_CO_3_ to H_2_O and CO_2_ is frozen by the strong bond formation between O_2_^−•^ and H_2_CO_3_. In our previous study,^3)^ the binding energy of O_2_^−•^····*trans*-trans H_2_CO_3_ was calculated to be 58.40 kcal mol^−1^ using highly accurate theoretical calculations (G3MP2B3).^3)^

### Field electron emission by the application of high-frequency voltage to the sharp metal needle

As shown in Scheme 1 in the ambient-air corona discharge, O_2_^−•^ as the intermediate ion is rapidly converted into CO_3_^−•^ as the terminal product ion *via* reactions (4)–(11). Thus, investigating the reactivity of O_2_^−•^ as the single reactant ion is difficult using corona discharge as the ion source. In fact, previous investigations of the reactions of O_2_^−•^ have primarily been conducted using flowing afterglow techniques.^23)^ However, we discovered that O_2_^−•^ was only formed as the product ion only when a high frequency voltage, but not a DC voltage, was applied to the needle electrode in the open ambient air (see Fig. 1(b)).

**Figure 4:**
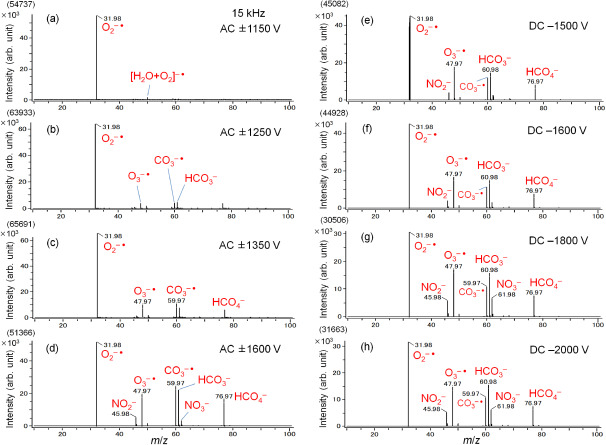
Fig. 4. Mass spectra obtained by the application of a 15 kHz AC voltage to the needle with (a) ±1150 V, (b) ±1250 V, (c) ±1350 V, and (d) ±1600 V. ±1150 V is the threshold voltage for the observation of ion signals. Mass spectra obtained by the application of DC voltage with (e) −1500 V, (f) −1600 V, (g) −1800 V, and (h) −2000 V. −1500 V is the threshold voltage for the observation of ion signals.

Figures 4(a)–4(d) show negative-mode mass spectra when a 15 kHz alternating current (AC) high voltage was applied to the needle. At the threshold voltage of ±1150 V for the detection of ion signals, O_2_^−•^ was detected as the product ion. If gas breakdown occurred with this voltage, O_3_ must be formed by the decomposition of O_2_ in the plasma (see Scheme 1(a)). The total absence of O_3_^−•^ (*m*/*z* 48) and CO_3_^−•^ (*m*/*z* 60) indicated that gas breakdown followed by the generation of a corona discharge did not occur at this threshold voltage of ±1150 V. The formation of O_2_^−•^ as the predominant ion suggests that *free* electrons were generated and they were converted into O_2_^−•^ in ambient air by the electron attachment reaction (4). It is likely that free electrons were generated by the field electron emission from the tip of the needle electrode. The conceptual scenario for the tunneling electron emission is depicted in Fig. S4. As shown in Fig. 4(b), O_3_^−•^ and CO_3_^−•^ started to be detected when the AC voltage was increased to ±1250 V. This suggests that the AC corona discharge started to contribute to ion formation in addition to field electron emission at ±1250 V. With a further increase in the AC high voltage to ±1600 V, NO*_x_^−^* ions (*x*=2, 3) originating from the decomposition of N_2_ (in Scheme 1(c)) were detected.

**Figure 5:**
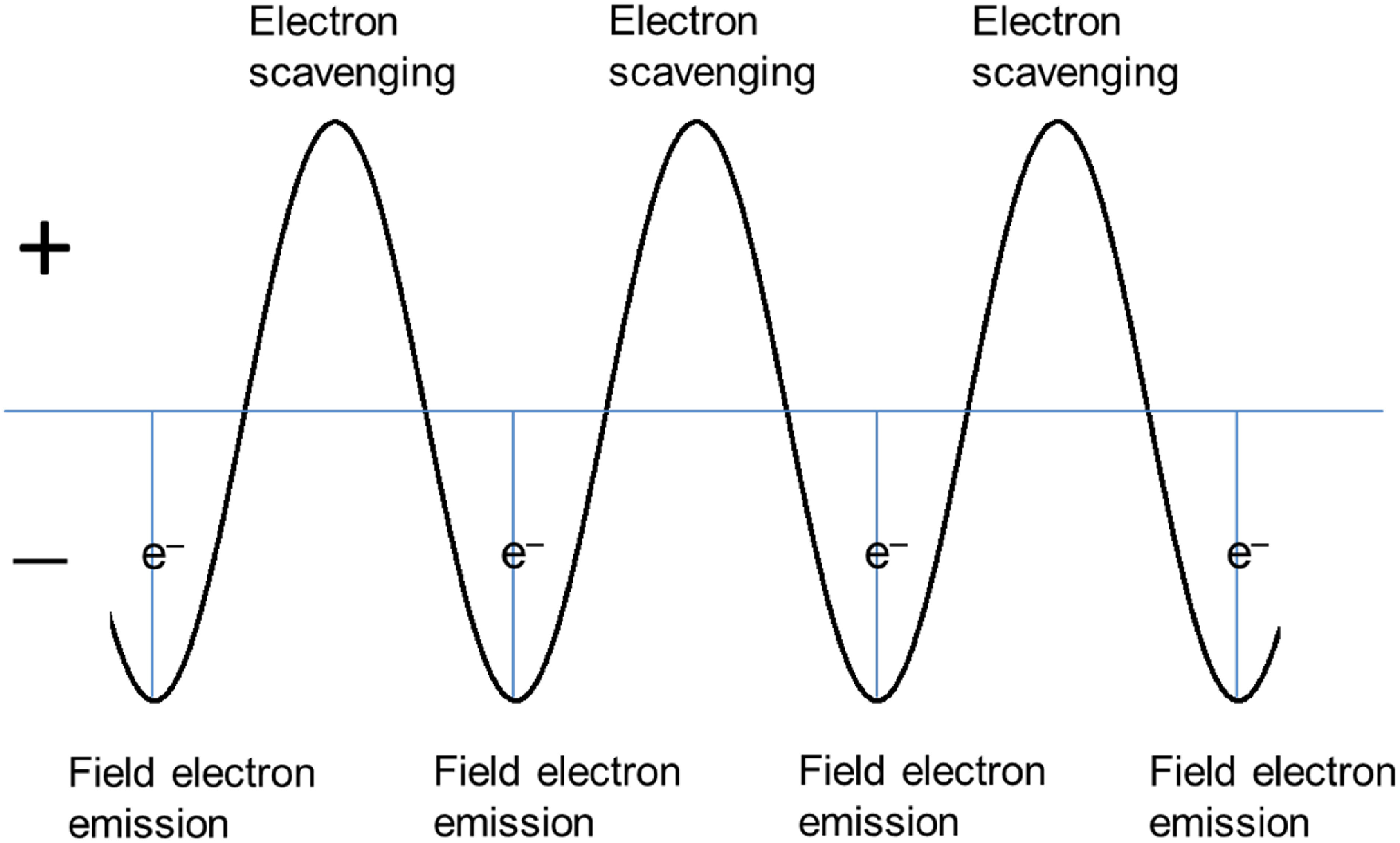
Fig. 5. An illustrative presentation of the tunneling electron emission when the threshold AC negative voltage was applied to a sharp needle electrode. In the positive phase of AC voltage, the emitted electrons are scavenged to the needle electrode making air restored as an insulating medium.

Figures 4(e)–4(h) show negative-mode mass spectra when a negative DC high voltage was applied to the needle. Figure 4(e) shows the mass spectrum when a threshold voltage of −1500 V was applied to the needle for the observation of ion signals. It should also be noted that the threshold voltage of −1500 V is considerably higher (more negative) than that of the negative-phase AC voltage of −1150 V shown in Fig. 4(a). It is apparent that in the DC mode of operation, field electron emission is largely suppressed and a corona discharge is directly generated at −1500 V. Furthermore, in the DC mode, the field electron emission must occur at around −1150 V at the precise moment of when a high voltage is applied to the needle. However, due to the continuous application of a negative DC high voltage to the needle, free electrons were emitted and O_2_^−•^ formed by electron attachment may have accumulated near the tip of the electrode. The accumulated negative charge near the tip of the needle should result in the formation of a space-charge field that shields the electric field at the tip of the needle. Due to the decrease in the electric field, the field strength at the needle tip becomes lower than that needed for the field electron emission. Such a build-up of the space charge field can be avoided by the application of an AC voltage because the free electrons and resulting O_2_^−•^ that are produced in the negative voltage phase can be completely scavenged by the metal needle by the subsequent positive phase high voltage that is applied, as shown in Fig. 5.

A separate experiment was performed to examine the electron scavenging effect suggested above. A high voltage pulse of −3000 V with a pulse width in the range of 200 ns to 100 μs was applied to the needle with and without the application of a positive bias voltage of +500 V. The pulse repetition rate was 20 Hz. With a pulse width of 200 ns, the ion signals started to be detected at −3000 V. Figs. S5(a)–S5(d) show mass spectra obtained with a bias voltage of 0 V. Even with a pulse width of 200 ns, discharge product ions such as NO_2_^−^ and CO_3_^−•^ were detected. With no bias voltage, the electrons emitted by the tunneling effect are accelerated by the strong electric field resulting in the breakdown of the gas. In contrast, only O_2_^−•^ was detected with a pulse width of 200 ns when a bias voltage of +500 V was applied, as shown in Fig. S5(e). It is evident that a bias voltage of +500 V is effective for scavenging electrons that are emitted by the tunneling effect and this suppresses the occurrence of corona discharge. However, a pulse width of 1 μs in Fig. S5(f) is sufficient for gas breakdown to occur as discharge product ions such as CO_3_^−•^ start to be detected.

The scavenging effect of the build-up of negative charges near the needle tip in the positive phase of AC high voltage should be dependent on the frequency of the AC high voltage. Fig. S6 shows mass spectra obtained when the frequency of the AC high voltage was changed in the range of 20 kHz to 5 kHz measured at the threshold voltage for the field electron emission. Cl^−^, HCOO^−^, and CH_3_COO^−^ product ions were formed by the reactions of O_2_^−•^ with HCl, formic acid, and acetic acid contaminants, respectively, that are present in the laboratory air (see the latter section). There seems to be no noticeable frequency dependence on the field electron emission in the range of 5–20 kHz.

In addition to the use of a stainless steel acupuncture needle, various other metal needles were tested as electrodes. Metal wire, with a diameter of 0.1 mm, was cut tangentially by a nipper and then sharpened using Emery paper (# 1000) and was used for an emitter. Among the tested metals (Ti, W, Cr, Co, Mo, Pt, Pd, Fe, Au, Ni, Ir, Cu, constantan (Cu/Ni alloy), Pd/Pt(1/9)), Ti, Pd, constantan, Pd/Pt(1/9), and Cr were found to be appropriate for field electron emission. There is a crude trend that metals with lower work functions are better-suited as field electron emitters.

**Figure 6:**
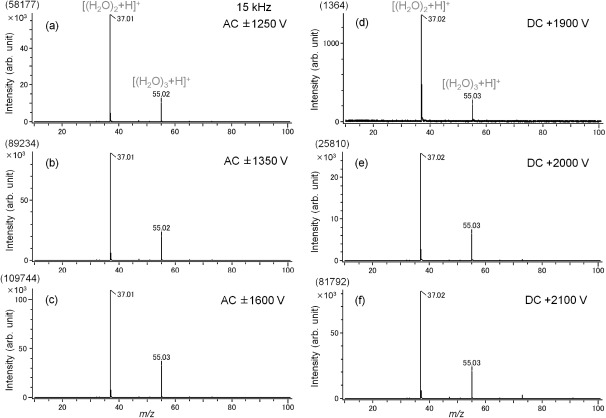
Fig. 6. Positive mode mass spectra measured by the application of AC ((a)–(c)) and DC high voltage ((d)–(f)). (a) and (d) show the mass spectra at the threshold voltage for the observation of ion signals for the application of AC and DC voltage, respectively.

In our previous paper, an AC corona discharge was applied to an atmospheric-pressure chemical ionization (APCI) ion source for the first time.^29)^ The AC corona discharge was found to be superior to a DC corona discharge for various reasons^3,29)^: (i) corrosion of the needle electrode by the AC corona is much less than that for a DC corona, (ii) both positive and negative ions can be detected without changing the polarity of the high voltage power supply, (iii) an AC corona gives as strong positive and negative ion signal intensities as a DC corona even though an intermittent plasma is generated in the AC corona, (iv) ionization by an AC corona is milder than that for a DC corona, (v) transition to arc discharge for an AC corona is largely suppressed compared to that for a DC corona. These characteristic differences between AC and DC corona discharges can be envisaged by the observation of positive-mode mass spectra obtained by AC and DC corona discharges. Figure 6 shows the positive-mode mass spectra for ambient air measured under the same experimental conditions as in Fig. 4. As suggested in Fig. 4(b), the corona discharge started at the threshold voltage of ±1250 V in the positive-mode for the AC corona discharge in Fig. 6(a). The signal intensities for [(H_2_O)*_n_*+H]^+^ (*n*=2, 3) increase only gradually with increasing AC voltage from ±1250 V to ±1600 V. In contrast, for the DC corona discharge as shown in Figs. 6(d)–6(f), the ion signal intensities increase steeply with increasing applied DC voltage from the threshold voltage of +1900 V to +2100 V. With a further increase in +DC high voltage, a transition to arc discharge was anticipated. It should be noted that the threshold voltage for the DC corona discharge (+1900 V) was much higher than that for the AC corona discharge (±1250 V). This indicates that AC and DC corona discharges are based on quite different breakdown mechanisms. Plasma is an electrically conducting media composed of positive and negative charges and is generated by an electron avalanche induced by electrons accelerated in a high electric field in an insulating media. In the positive-mode DC discharge, nascent electrons that trigger the discharge are generated accidentally by cosmic rays or photoelectrons. The greater fraction of the nascent electrons are attracted to the anode, where they are annihilated by the high electric field near the anode. Due to the paucity of electrons that act as the seeds for gas discharge breakdown, a high threshold voltage is necessary for initiating a positive-mode DC corona discharge. In addition, the incidental generation of nascent electrons may lead to the positive-mode corona discharge being unstable. Owing to the application of a positive potential to the anode for the positive-mode DC corona, positive ions accumulate near the tip of the needle electrode leading to the formation of a Debye sheath that shields the potential applied to the needle. This explains why a high positive potential is necessary for maintaining a stable positive-mode DC corona discharge. In contrast, in the AC corona discharge, electrons are supplied to the insulating medium by the field electron emission in the negative-phase voltage, enabling the maintenance of discharge with a much lower applied voltage. In summary, the AC corona discharge can be maintained with a much lower voltage than DC corona discharge for both positive- and negative-mode of mass spectrometric operation.

A negative-mode corona discharge is beneficial for the detection of molecules that have positive electron affinities because all electrons are eventually converted into negative ions by electron attachment reactions such as the formation of O_2_^−•^ in this experiment.

**Figure 7:**
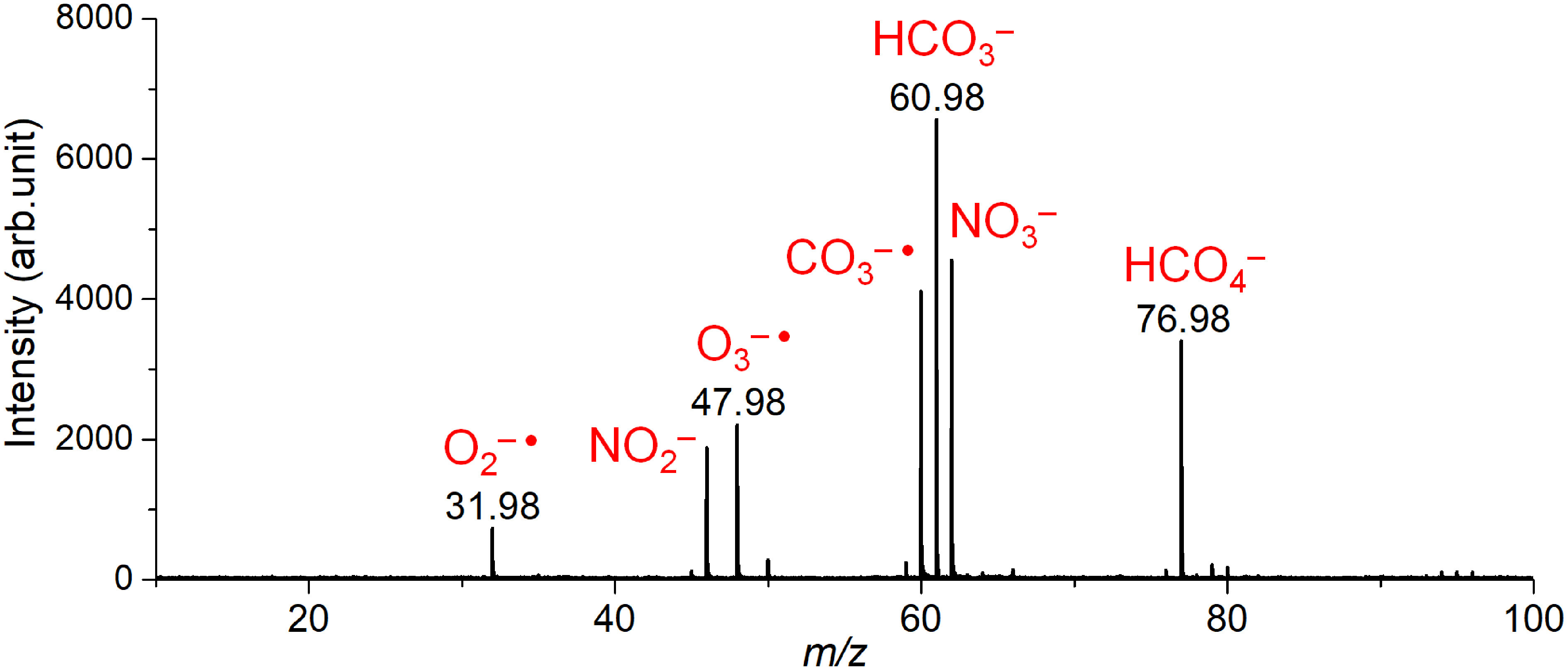
Fig. 7. Mass spectrum for the plasma-excited air generated by the commercial air sterilizer.

As described in the introduction, CO_3_^−•^ causes oxidative damage to biological systems such as DNA and proteins. As shown in Fig. 4, a corona discharge generates reactive oxidants of O_2_^−•^, CO_3_^−•^, O_3_, *etc*. There are many commercially available household appliances that use a corona discharge for the sterilization of bacteria and virus in air. To examine the kinds of ions that are formed by sterilizers that use a corona discharge, the plasma-activated air flowing out from a commercial air sterilizer (USB type, Air Success Mini, Air Success, Kanagawa, Japan) was measured. Figure 7 shows a mass spectrum for air ionized by the negative DC mode multiple-ring corona discharge that is installed in the Air Success Mini. The mass spectrum is very similar to those shown in Figs. 4(e)–4(h) and oxidative CO_3_^−•^ was detected as one of the major ions.

### Reactions of O_2_^−•^ with various molecules

**Figure 8:**
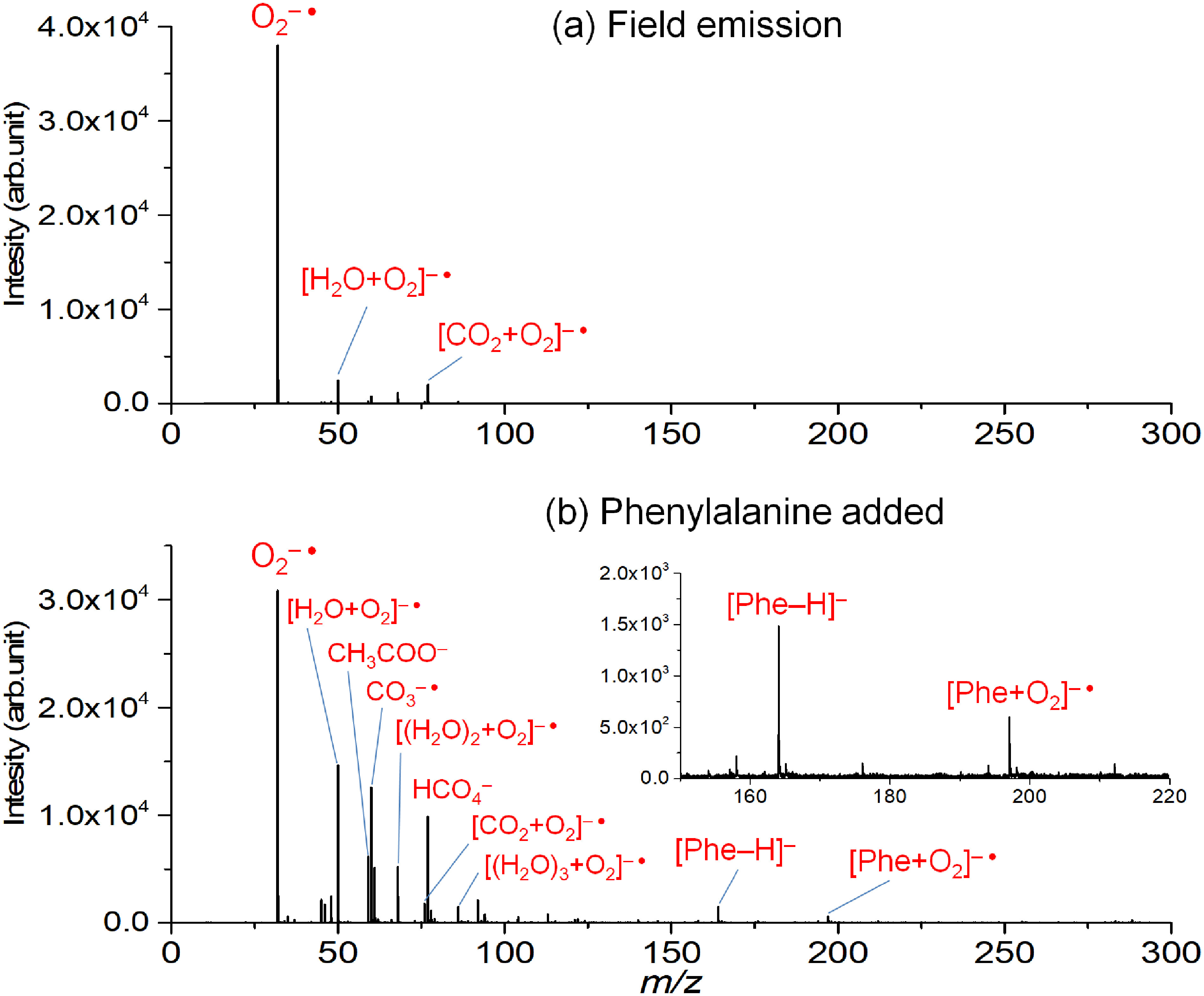
Fig. 8. (a) Mass spectrum obtained before sample introduction. (b) Mass spectrum showing the introduction of phenylalanine (Phe) into the ion source. A 10 μL aliquot of a 10^−3^ M aqueous solution of phenylalanine was placed on the heater. At 10 s after the sample was placed on the heater at 140°C, the sample began to gradually evaporate.

The reactions of O_2_^−•^ with hydrocarbons, alcohols, acetone, and acetonitrile were examined by placing 10 μL liquid samples in the heater shown in Fig. 1(b). Neither H^•^ nor H^+^ abstraction reactions were observed for these compounds. However, when 10 μL formic acid, acetic acid, and trifluoroacetic acid were introduced into the ion source, the respective deprotonated carboxylate ions HCOO^−^, CH_3_COO^−^, and CF_3_COO^−^ were clearly detected as the major ions. After these three measurements, a mass spectrum for laboratory air contaminated by these three acids was collected, as shown in Fig. S7. All three acids were clearly detected indicating that the present field-electron-emission type ion source is suitable for the detection of trace amounts of acids. Deprotonated ions were also detected for several amino acids (leucine, isoleucine, alanine, and phenylalanine). Figures 8(a) and 8(b) show the mass spectra before and after introducing phenylalanine (Phe) into the ion source. Approximately 10 s after the deposition of a 10 μL aqueous solution of a 10^−3^ M phenylalanine on the heater at 140°C, deprotonated [Phe−H]^−^ and [Phe+O_2_]^−•^ cluster ions started to be detected. The ion signals continued to be detected for much longer than 10 s due to the slow evaporation of the phenylalanine at 140°C (melting point: 283°C, boiling point: 295°C). Fig. S8 shows the results obtained for nitric acid. Fig. S8(a) shows the mass spectrum before sample introduction, in which O_2_^−•^ is detected as the only major ion. Fig. S8(b) shows the mass spectrum obtained when a cotton ball that was wetted by a 30% aqueous nitric acid was positioned in close proximity to the ion source at 0.24 min. O_2_^−•^ was completely converted into NO_3_^−^ and [HNO_3_+NO_3_]^−^. Figs. S8(c)–S8(e) show EIC for O_2_^−•^, NO_3_^−^, and [HNO_3_+NO_3_]^−^, respectively. NO_3_^−^ was detected for hours after the sample introduction.

Table S2 summarizes the enthalpy changes for proton transfer reactions of O_2_^−•^, HCO_3_^−^, and CO_3_^−•^ with acetic, formic and nitric acids.^25,26)^ While all reactions are exothermic for O_2_^−•^, those for HCO_3_^−^ and CO_3_^−•^ are endothermic except for nitric acid. This is due to the fact that the proton affinity of O_2_^−•^ (353 kcal mol^−1^) is much larger than that for CO_3_^−•^ (333 kcal mol^−1^) and of HCO_3_^−^ (338.7 kcal mol^−1^)^25,26)^ (see Scheme 1).

## CONCLUSION

In gas-phase reactions of CO_3_^−•^, O^−•^ transfer, O_2_^−•^ transfer, and H^+^ abstraction reactions with inorganic and organic molecules have been studied to date. However, H^•^ abstraction reactions with organic molecules, although of interest, have not been reported. In this work, occurrence/nonoccurrence experiments of H^•^ abstractions of CO_3_^−•^ with various molecules in the gas phase are reported for the first time. H^•^ abstraction was observed for *n*-hexane, cyclohexane, methanol, ethanol, 1-propanol, 2-propanol, and toluene, but no reactions were observed for acetonitrile, acetone, benzene, and H_2_O. DFT calculations clearly demonstrated the reason for this contrast between the occurrence for toluene and *n*-hexane and the nonoccurrence for benzene. In biological systems, CO_3_^−•^ is capable of causing serious oxidative damage to proteins and DNA molecules *via* H^•^ abstraction reactions. It should therefore be assumed that air sterilizers with the function *via* the use of a corona discharge ion source evolve CO_3_^−•^ ions as the major ions, which could be harmful to mucous membranes such as lungs.

When an AC high voltage was applied to the sharp metal needle electrode in ambient air, tunneling electron emission from the tip of the needle was observed and the generation of electrons were detected as O_2_^−•^ by an electron attachment reaction. O_2_^−•^ did not show any reactivity toward hydrocarbons or alcohols but it abstracts H^+^ from acid molecules such as formic acid, acetic acid, nitric acid and amino acids. By investigating the threshold behavior of ion formation for AC and DC corona discharges, the reason why an AC corona is milder than a DC corona has been elucidated.

## Notes

Mass Spectrom (Tokyo) 2021; 10(1): A0100
