# Supplementary material for: Corona Discharge and Field Electron Emission in Ambient Air Using a Sharp Metal Needle: Formation and Reactivity of CO3−• and O2−•
Source: Mass Spectrom (Tokyo). 2021 Dec 25;10(1):A0100. doi: 10.5702/massspectrometry.A0100 (PMC8697365; doi:10.5702/massspectrometry.A0100)
Supplement: Supplementary Data [file massspectrometry-10-1-A0100_s001.pdf]

Corona Discharge and Field Electron Emission in Ambient Air Using a Sharp Metal Needle: Formation and Reactivity of $CO_3^{-\bullet}$ and $O_2^{-\bullet}$

Kenzo Hiraoka, Stephanie Rankin-Turner, Satoshi Ninomiya, Haruo Shimada, Kazumasa Kinoshita, Shinichi Yamabe

Supporting Information

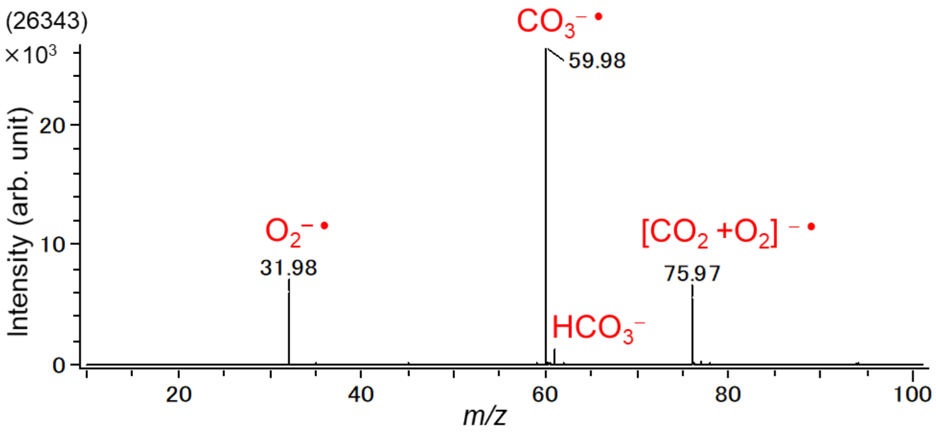

Fig. S1. Mass spectrum obtained when 99.9% CO_2 reagent gas was used using the discharge tube shown in Fig. 1(a). Flow rate of CO_2 gas: 3L min^{-1}. The peak at *m/z* 61 was identified as HCO_3^- because the intensity ratio of [*m/z* 61]/[*m/z* 60] (0.05) is larger than the isotope ratio of $^{13}C/^{12}C$=0.01.

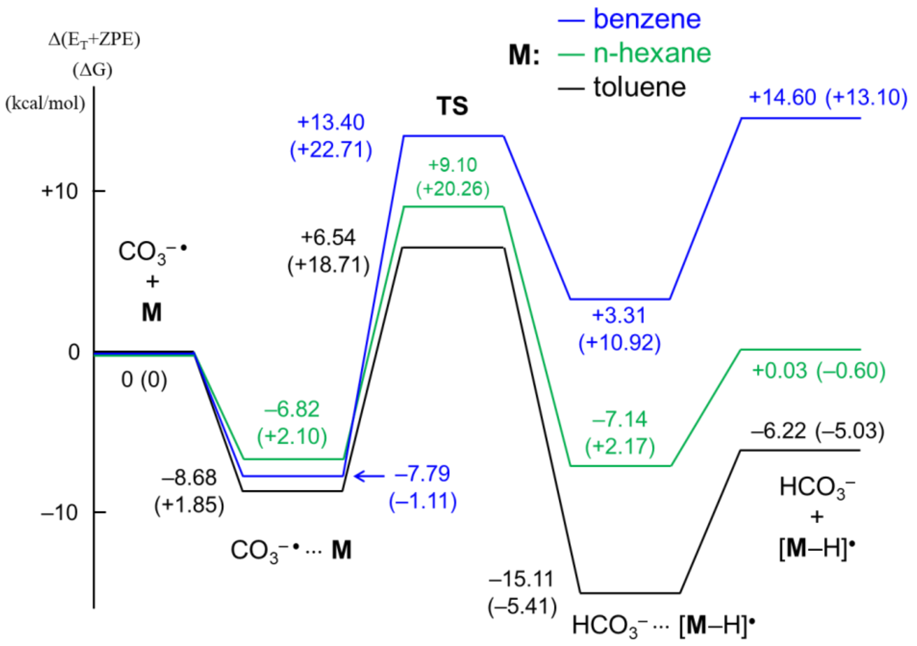

Fig. S2. Energy diagrams of the reaction, $CO_3^{-\bullet} + M \rightarrow HCO_3^- + [M\text{–}H]^\bullet$, obtained by M06-2X/6-311++G(2d,p) calculations. TS geometries are shown in Fig. S3. For *n*-hexane, three reactions leading to $H_2C^\bullet$–$(CH_2)_4$–CH_3, H_3C–$CH^\bullet$–$(CH_2)_3$–CH_3 and H_3C–CH_2–$CH^\bullet$–$(CH_2)_2$–CH_3 are possible. The first one with the terminal radical center is unstable. On the other hand, the latter two are formed via almost the same energy changes. The reaction leading to H_3C–CH_2–$CH^\bullet$–$(CH_2)_2$–CH_3 was adopted.

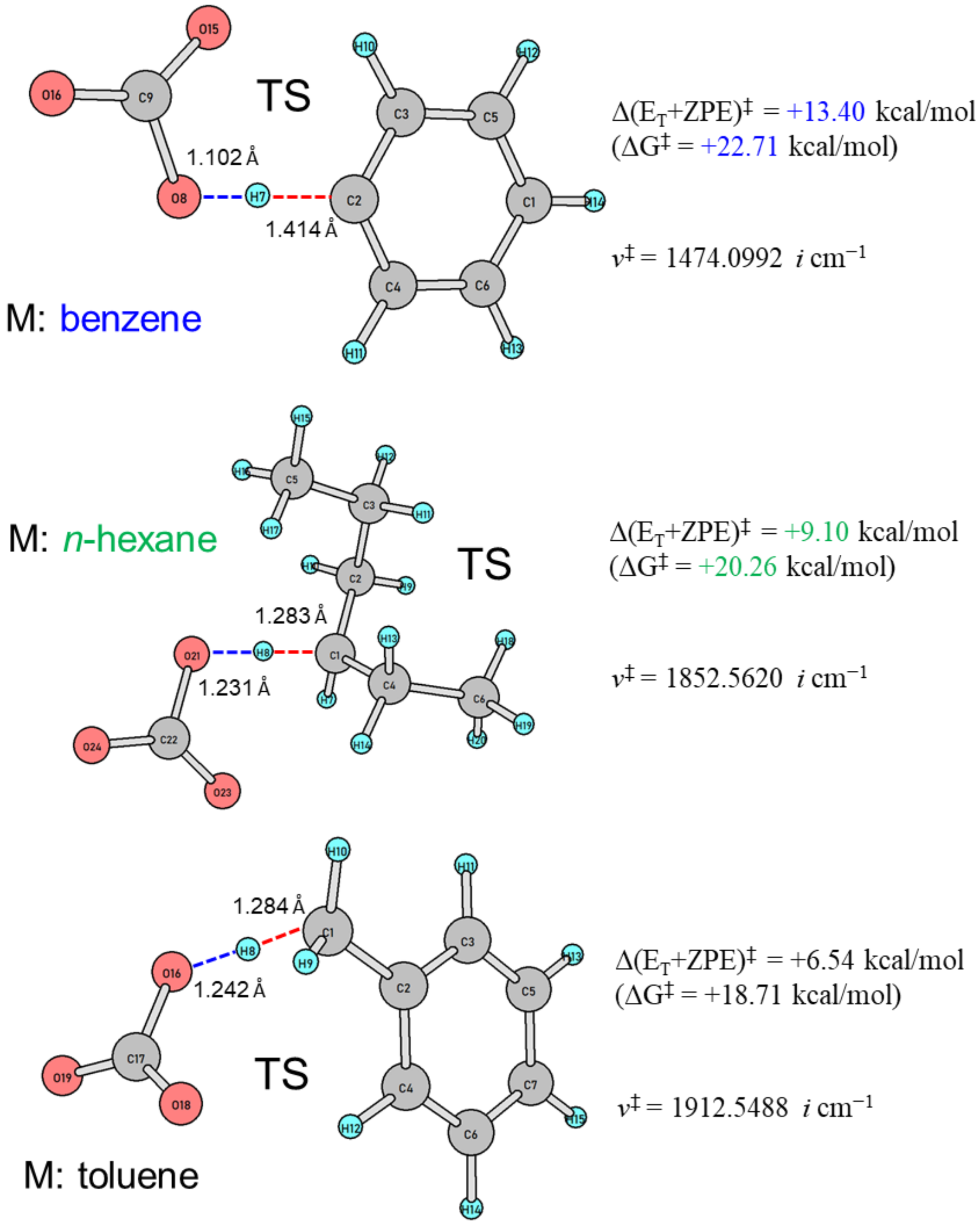

Fig. S3. TS geometries of the hydrogen-atom migration. Red and blue-color broken lines stand for covalent bonds cleaved and formed, respectively.

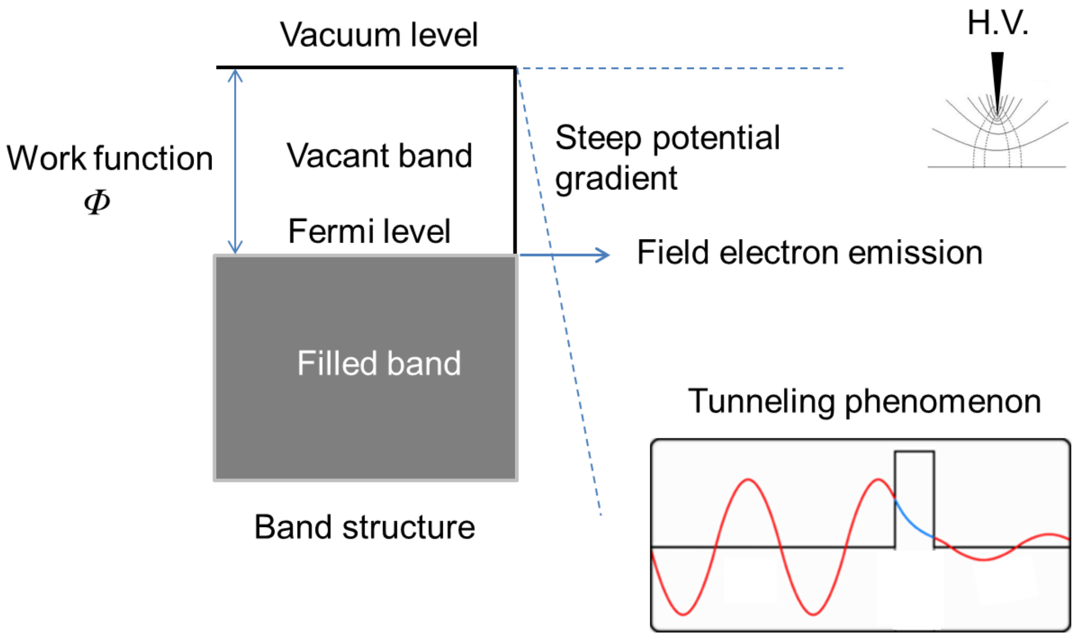

Fig. S4. The conceptual idea for the tunneling electron emission. Free electrons in the filled band of the metal penetrate through the barrier due to the wave-like nature of electrons.

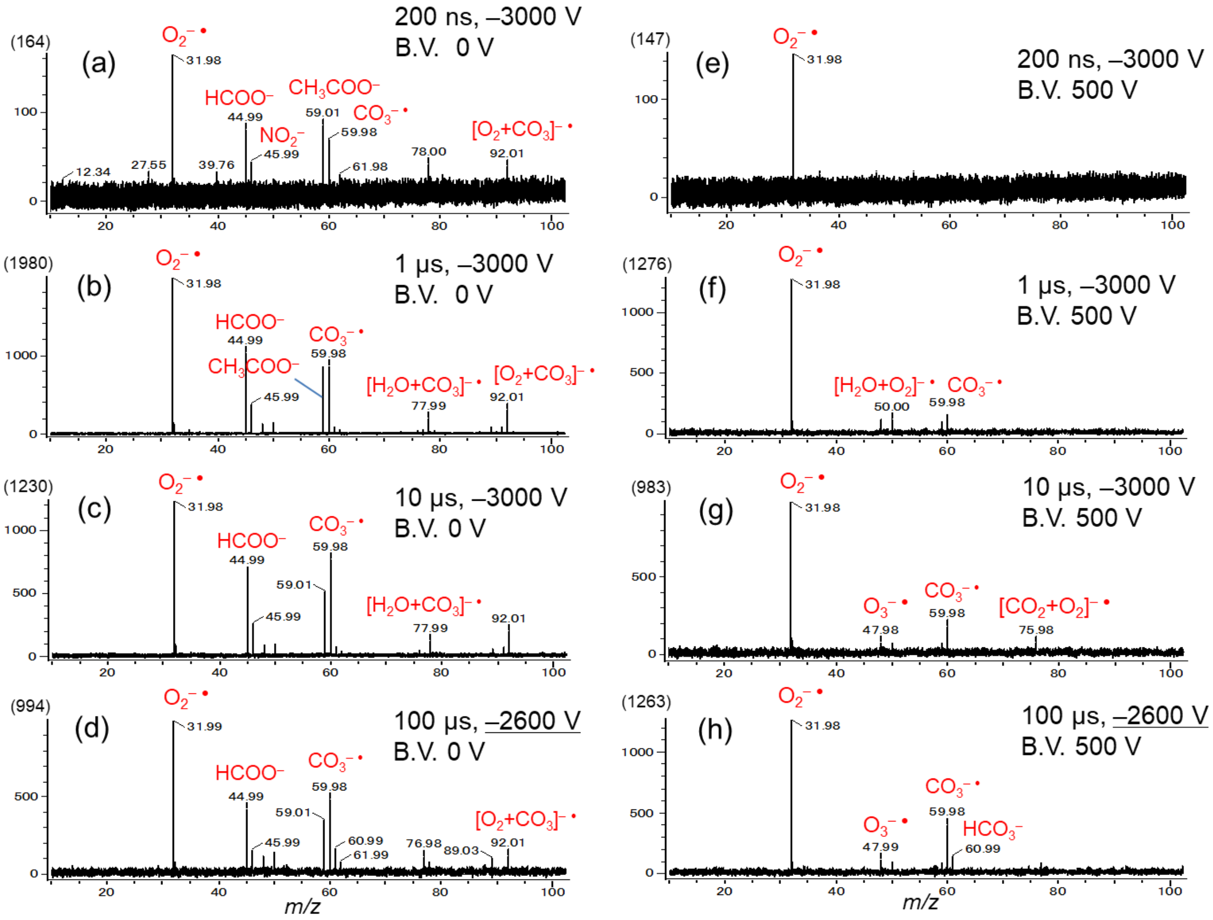

Fig. S5. Mass spectra measured by the application of a pulse voltage of –3000 V to the acupuncture needle in ambient air with pulse widths in the range of 200 ns to 100 μs. (a)–(d) bias (offset) voltage: 0 V. (e)–(f): bias voltage: +500 V. A pulse voltage of –3000 V is the threshold for the observation of ion signals with a pulse width of 200 ns.

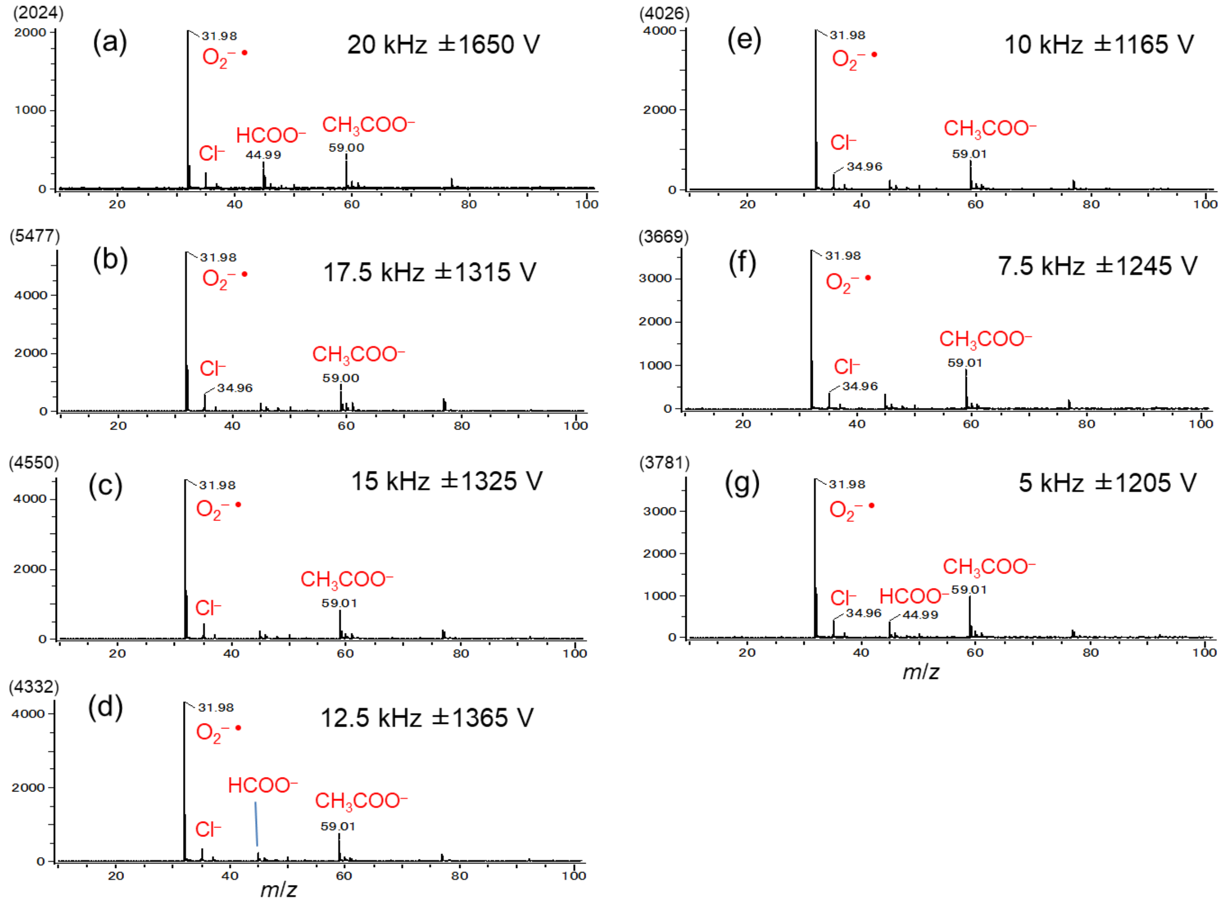

Fig. S6. Mass spectra obtained when the frequency of the AC high voltage was changed in the range of 20 kHz to 5 kHz measured at the threshold voltage of field electron emission.

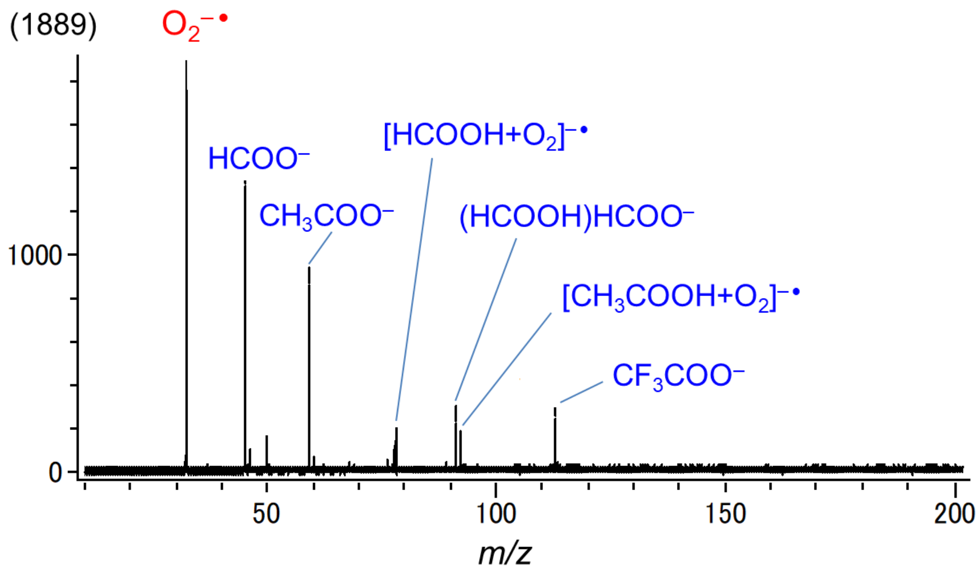

Fig. S7. Mass spectrum for laboratory air contaminated by formic, acetic and trifluoroacetic acid vapors obtained at the threshold AC voltage of ±1150 V.

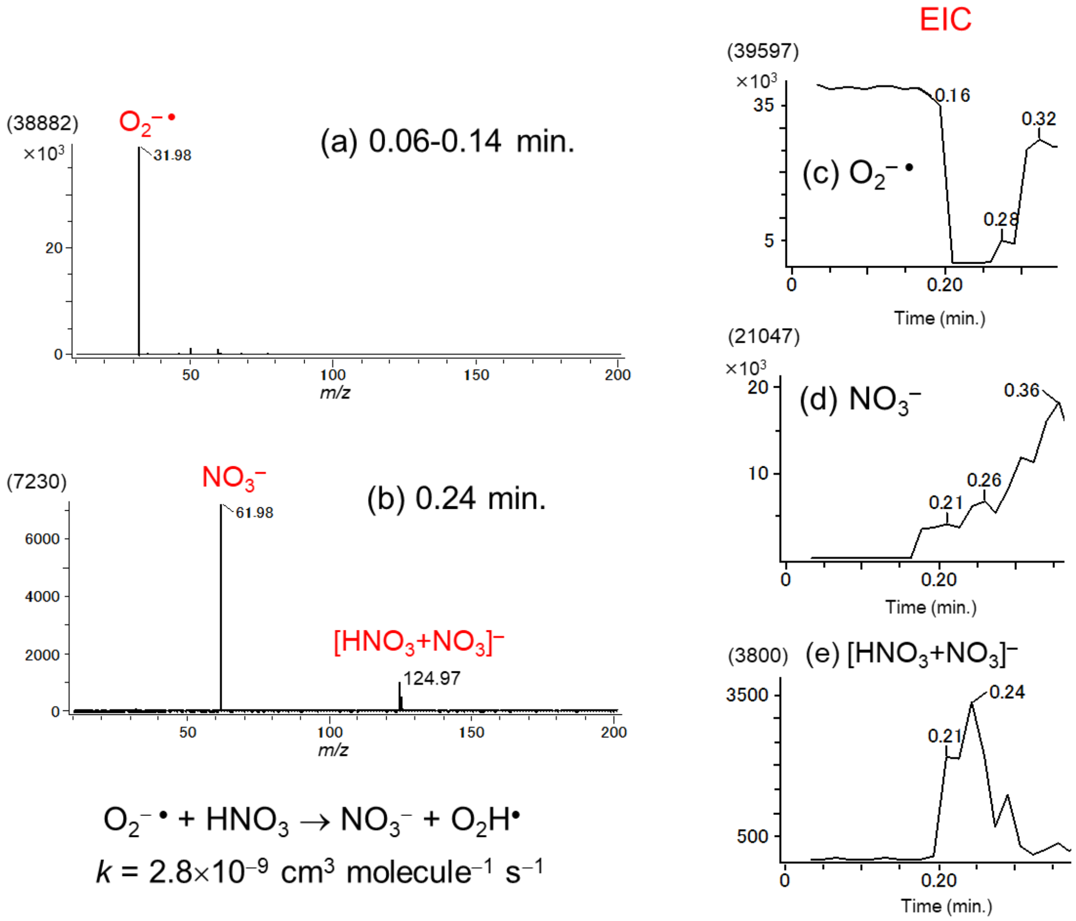

Fig. S8. (a) Mass spectrum before sample introduction. (b) mass spectrum when a cotton bud wetted by 30 % aqueous nitric acid was positioned in close proximity to the ion source at 0.24 min. (c)–(e): EIC for $O_2^{-\bullet}$, NO_3^-, and $[HNO_3 + NO_3]^-$, respectively.

Reactions of $CO_3^{-\bullet}$

	bond energy	$H^{\bullet}$ abstraction
Methanol	94.2	observed
Ethanol	92.3	observed
Acetonitrile	93.2	not observed
n-hexane	99.1	observed
Cyclohexane	99.7	observed
Toluene	89.2	observed
Acetone	96.5	not observed
Benzene	111.4	not observed
H_2O	119.3	not observed

$BE(H^{\bullet}\cdots CO_3^-)$: 105.7 kcal mol^{-1}

Table S1. Bond energies of H–C and H–O bonds for compounds which are available in refs. (25) and (26).

Proton transfer reaction

$O_2^{-\bullet}$

$O_2^{-\bullet} + CH_3COOH \rightarrow HO_2^{\bullet} + CH_3COO^-$	–4.6 kcal/mol
$O_2^{-\bullet} + HCOOH \rightarrow HO_2^{\bullet} + HCOO^-$	–7.8 kcal/mol
$O_2^{-\bullet} + HNO_3 \rightarrow HO_2^{\bullet} + NO_3^-$	–28.6 kcal/mol

HCO_3^-

$HCO_3^- + CH_3COOH \rightarrow H_2CO_3 + CH_3COO^-$	10.1 kcal/mol
$HCO_3^- + HCOOH \rightarrow H_2CO_3 + HCOO^-$	6.9 kcal/mol
$HCO_3^- + HNO_3 \rightarrow H_2CO_3 + NO_3^-$	–13.8 kcal/mol

$CO_3^{-\bullet}$

$CO_3^{-\bullet} + CH_3COOH \rightarrow HCO_3^{\bullet} + CH_3COO^-$	15.8 kcal/mol
$CO_3^{-\bullet} + HCOOH \rightarrow HCO_3^{\bullet} + HCOO^-$	12.6 kcal/mol
$CO_3^{-\bullet} + HNO_3 \rightarrow HCO_3^{\bullet} + NO_3^-$	–8.1 kcal/mol

Table S2. Heats of reaction values (kcal mol^{-1}) for proton transfer reactions of $O_2^{-\bullet}$, HCO_3^-, and $CO_3^{-\bullet}$ with three acid molecules, CH_3COOH, $HCOOH$, and HNO_3.
